# Supplementary material for: Early empathy development: Concern and comforting in 9- and 18-month-old infants from Uganda and the UK
Source: PLoS One. 2025 May 2;20(5):e0320371. doi: 10.1371/journal.pone.0320371 (PMC12047753; doi:10.1371/journal.pone.0320371)
Supplement: S1 File — Including coding scheme, full model outcomes, and additional analyses. (DOCX) [file pone.0320371.s001.docx]

**Supplementary Materials**

**S1 Overview of Available Data**

Table S1 shows the total number of participants tested at each age and at each site, alongside the number of valid trials for each age group, population, and condition. Exclusions were made based on: mother errors while simulating distress (15 trials excluded in Uganda, 16 in the UK; no trials had to be excluded due to experimenter errors); participant inattentiveness or distress (2 trials in Uganda, 4 trials in the UK but all overlapped with exclusions due to adult simulation errors); or interference of other individuals (1 trial in Uganda). In Uganda, out of the 40 infants who completed the experiment at 9 months, 34 returned at 18 months and 4 new participants were added to the sample. In the UK, 42 of 48 participants remained in the sample from 9 to 18 months and 1 new participant was added at 18 months. In Uganda, 32 infants had valid experimental trials with both the mother and the experimenter, 3 had a valid trial only with the mother and 2 had a valid trial only with the experimenter. In the UK, 39 infants had valid experimental trials with both the mother and the experimenter, 1 had a valid trial only with the mother and 3 had a valid trial only with the experimenter.

**Table S1**

**Number of Participants Tested by Age, Population, and Condition**

| Age |  | Number of Participants Tested | Number of Valid Trials | | |
| --- | --- | --- | --- | --- | --- |
|  | Population |  | Control | Experimental | Total |
| 9 months | Uganda | 40 | 36 | 38 | 74 |
|  | UK | 48 | 44 | 46 | 90 |
| 18 months (mother) | Uganda | 37 | 35 | 35 | 70 |
|  | UK | 43 | 38 | 40 | 78 |
| 18 months (experimenter) | Uganda | 37 | 35 | 34 | 69 |
|  | UK | 43 | 41 | 42 | 83 |
| Total |  |  | 229 | 235 | 464 |

**S2 Bluetooth Earpiece Instructions**

**Table S2**

***Experimental Condition Instructions Given to Mothers and Experimenters^1^ (Scenarios A and B)***

| Time | Instruction |
| --- | --- |
| 1.00 | Say "Oooh" and "Ouch"^2^ as if it hurts whilst rubbing your finger^3^. Look at your finger^3^. |
| 4.50 | 7 sec interval^4^ |
| 11.50 | Continue saying "Ouch" whilst rubbing your finger^3^. Alternate looking at your finger^3^ and your child's face. |
| 16.00 | 7 sec interval^4^ |
| 23.00 | Continue rubbing your finger^3^ but stop saying "Ouch". |
| 25.50 | 1 second interval^4^ |
| 26.50 | Look at your child and say "Look, I hurt myself". |
| 29.00 | 3 sec interval^4^ |
| 32.00 | Look back at your finger^3^ and keep your gaze on it. |
| 34.00 | 2 sec interval^4^ |
| 36.00 | Wonderful, thank you! You can now play with your child. |

*Note*. ^1^Instructions given via Bluetooth earpiece for experimenters and UK mothers. Equivalent instructions were given in an appropriate language verbally by a researcher wearing the earpiece for Ugandan mothers (procedure described in main MS). ^2^In Uganda, mothers were instructed to give culturally appropriate nonverbal pain vocalisations. ^3^In scenarios C and D, the “finger” was replaced by “knee” and “foot” respectively. ^4^The intervals were periods of silence on the recording.

**S3 Ethogram for Video Coding**

| Ethogram used for video coding in ELAN of control and experimental condition for 9- and 18-month-olds and for mother and experimenter trials based on Zahn-Waxler et al. (1992). Within a tier of behaviours (e.g., visual attention), categories were mutually exclusive. All categories were coded on a start-and-stop basis for the duration of the behaviour.  **Table S3**  **Full Coding Scheme of the Comforting Task**  **Visual Attention to Distress** | |
| --- | --- |
| Visual attention coding is applied before comforting coding, as a minimum of visual attention to the distressed adult model is a prerequisite for behaviours to be considered comforting. | |
| Category | Description |
| Face | Looking at distressed model’s face |
| Body | Looking at distressed model’s hurt body part. Can be hand in general if finger is hurt or the corresponding limb (e.g., looking at left knee when right knee is hurt) |
| Other | Looking at another individual, e.g., sibling, non-model adult, second experimenter behind camera |
| None | Does not look at distressed or other individual, may look up/down, at object, own body. |
| **Comforting** | |
| Behaviours are only considered comforting if the infant looked at the distressed adult model’s face/the hurt body part (see visual attention coding) within the 3s before the behaviour onset. | |
| Category | Description |
| Pat/Stroke | Gentle movement of the infant’s hand on the distressed model’s body.  NOT hitting, pulling hair, pointing (with finger touching model) |
| Hug | Extended arms clasping the distressed model while the infant’s torso is in contact with the model.  NOT pulling self up on model body, leaning sideways against model, lying face down on lap/legs |
| Kiss | Touch the distressed model’s face, hurt body part or other limb with the mouth. NOT falling into the adult head first. |
| Object | Voluntarily hand the distressed model an object (NOT toy hammer/clipboard). Code regardless of model (not) picking it up. |
| Verbal | Vocalisations indicating concern such as “You okay?,” “Oh oh!”, "Ouch!” Note that vocalisations (e.g., “Ouch?”) with questioning intonation are coded as verbal hypothesis testing. |
| None | No observable comforting behaviours |
| **Child Affect** | |
| Category | Description |
| Distress | Crying, whimpering, whining, fussing |
| Concern | Eyebrows raised and/or drawn together, eyes widened, frowning, tight lips, not paired with distress vocalisations (see above) |
| Not Visible | Face is not visible from either camera angle |
| None/Other | Infant facial expression is visible but neutral or does not match either distress of concern description |
| **Hypothesis Testing** | |
| Category | Description |
| Nonverbal | E.g., touches equivalent body part, copies action, looks back and forth from distressed model to caregiver/experimenter, looks back and forth from face to hurt body part |
| Verbal | Vocalisations with questioning intonation (“Huh?,” “Ouch?”) while paying attention to adult distress, questions about model’s distress, e.g. “What happened?” |
| None | No observable hypothesis testing behaviours |

**S4 Model Checks**

All models were checked for collinearity issues using Variance Inflation Factors (VIF), performed on standard linear models with no interactions and no random effects. For beta GLMMs, we assessed overdispersion using a function provided by Roger Mundry. We found no issues of collinearity or overdispersion (all VIF between 1 and 1.01; maximum dispersion parameter = 1.31). Model stability was assessed using dfbeta for GLMs, by using the function glmm_stability for binomial GLMMs and by using the function glmmTMB_stability for beta GLMMs. For GLMs, confidence intervals were calculated via bootstrapping using the function predict.glm. For binomial GLMMs, confidence intervals were derived through bootstrapping using the function bootMer of the package lme4, and through simulation using the function simulate of the package glmmTMB for beta GLMMs.

**S5 Specific Comforting Behaviours**

**Table S5**

**Absolute Frequencies of Specific Comforting Behaviours in the Experimental Condition by Population, Age, and Model Familiarity.**

|  | Uganda | | | UK | | |
| --- | --- | --- | --- | --- | --- | --- |
|  | 9 months | 18 months | | 9 months | 18 months | |
|  | Mother | Mother | Experimenter | Mother | Mother | Experimenter |
| Number of Valid Trials | 38 | 35 | 34 | 46 | 40 | 42 |
| Pat | 4 | 8 | 3 | 4 | 5 | 0 |
| Hug | 2 | 3 | 0 | 0 | 2 | 0 |
| Kiss | 0 | 1 | 0 | 0 | 0 | 0 |
| Hand Object | 1 | 1 | 1 | 0 | 3 | 0 |
| Verbal | 0 | 0 | 0 | 0 | 1 | 3 |

**S6 Additional Analysis: Hypothesis Testing**

We coded frequencies of inquiry behaviour or ‘hypothesis testing’, i.e., attempts by the infant to understand the adult model’s distress, based on Zahn-Waxler et al. (1992). The following behaviours were coded: the infant touching the equivalent body part on their own body to the one the model had hurt, imitating their distress signals (e.g., rubbing their own foot or finger, equivalent to the one the adult has hurt), or gazing back and forth between the distressed model’s face and the hurt body part. Infant vocal distress was not considered an imitation of model distress signals and therefore not coded as hypothesis testing. We extracted binary scores for infants displaying hypothesis testing during a trial. We opted for occurrence rather than duration or intensity, as longer orientation and attention towards the model in distress may be due to either less understanding of the distress (necessitating more hypothesis testing) or better understanding (reducing hypothesis testing). Reliability was good with Cohen’s kappa at 0.78.

Although we treat hypothesis testing with caution considering its reliance on coding of gaze alternation which may have been increased due to the distressed model’s gaze patterns in our particular design, below we present the results for the analysis of hypothesis testing. Model structures were equivalent to those used for the occurrence of comforting. Model tables below are for the reduced (main effects only) models which had better fit than the full model with an interaction term.

**Condition Effect**

The full-null model comparison was significant (*χ^2^*(5) = 148.63, *p* < .001) but the reduced model comparison was not (*χ^2^*(2) = 3.24, *p* = .198). Infants were more likely to show hypothesis testing in the experimental than in the control condition (estimate±SE = 2.41 ± 0.30, *χ^2^*(1) = 97.08, *p* < .001; Table S6a).

**Table 6a**

**Model Estimates with Standard Errors, Confidence Intervals, and Test Results**

| Term | Estimate | SE | Lower CI | Upper CI | *χ^2^* | df | p |
| --- | --- | --- | --- | --- | --- | --- | --- |
| (Intercept) | -3.41 | 0.39 | -4.31 | -2.82 |  |  | ^(1)^ |
| Condition (experimental) | 2.41 | 0.30 | 1.93 | 3.08 | 97.08 | 1 | <.001 |
| Age (18 months) | 2.03 | 0.32 | 1.53 | 2.73 | 55.69 | 1 | <.001 |
| Population (UK) | -0.53 | 0.25 | -1.07 | -0.07 | 4.66 | 1 | .031 |
| Sex (male) | -0.37 | 0.25 | -0.86 | 0.11 | 2.19 | 1 | .139 |

^(1)^ H_0_ for the intercept is *intercept = 0.* The intercept represents the hypothetical value of the response assuming all predictors and random effects are zero, but the absolute baseline of the response is not of interest here, so this is not reported.

**Age and Site Effects**

The full-null model comparison was significant (*χ^2^*(3) = 37.29, *p* < .001) but the full-reduced model comparison was not (*χ^2^*(1) = 0.32, *p* = .574). Infants’ likelihood to show hypothesis testing increased significantly with age (estimate±SE = 2.33 ± 0.53, *χ^2^*(1) = 36.48, *p* < .001) but there was no effect of site (estimate±SE = -0.30 ± 0.42, *χ^2^*(1) = 0.54, *p* = .463; Table 6b).

**Table 6b**

**Model Estimates with Standard Errors, Confidence Intervals, and Test Results**

| Term | Estimate | SE | Lower CI | Upper CI | *χ^2^* | df | p |
| --- | --- | --- | --- | --- | --- | --- | --- |
| (Intercept) | -1.22 | 0.43 | -10.56 | -0.48 |  |  | ^(1)^ |
| Age (18 months) | 2.33 | 0.54 | 1.64 | 21.81 | 36.48 | 1 | <.001 |
| Population (UK) | -0.30 | 0.42 | -1.21 | 0.49 | 0.54 | 1 | .463 |
| Sex (male) | -0.93 | 0.44 | -2.23 | -0.14 | 4.93 | 1 | .026 |

^(1)^ Not indicated because of having a very limited interpretation – see Table 6a for explanation

**Familiarity and Site Effects**

The full-null model comparison did not reach significance (*χ^2^*(3) = 4.35, *p* = .226), indicating that infants at both sites showed hypothesis testing at similar levels towards their mothers and the experimenters. The results of the full model are presented in Table 6c.

**Table 6c**

**Model Estimates with Standard Errors, Confidence Intervals, and Test Results**

| Term | Estimate | SE | Lower CI | Upper CI | *χ^2^* | df | p |
| --- | --- | --- | --- | --- | --- | --- | --- |
| (Intercept) | 1.62 | 0.46 | 0.83 | 2.85 |  |  | ^(1)^ |
| Adult (mother) | -0.84 | 0.55 | -2.08 | 0.22 |  |  | ^(2)^ |
| Population (UK) | -0.96 | 0.53 | -2.21 | 0.02 |  |  | ^(2)^ |
| Sex (male) | -0.56 | 0.35 | -1.34 | 0.16 | 2.60 | 1 | .107 |
| Adult (mother) * population (UK) | 0.86 | 0.72 | -0.62 | 2.40 | 1.47 | 1 | .225 |

^(1)^ Not indicated because of having a very limited interpretation - see Table 6a for explanation ^(2)^ Not indicated due to involvement in interaction

**S7 Model Estimates**

Where a reduced model (no interaction terms) was used to assess main effects of fixed effect variables (i.e., because an interaction term from the full model was not significant and had been removed), the model estimates of this reduced model, not the full model, are presented. Binary variables were dummy coded, with the reference category indicated in parentheses.

**Condition Effect**

***S7a Concerned Facial Affect***

Results of the reduced beta model of the effects of condition, age, population, and infant sex on proportion of visible trial time infants spent showing concerned facial affect can be found in Table S7a.

**Table S7a**

**Model Estimates with Standard Errors, Confidence Intervals, and Test Results**

| Term | Estimate | SE | Lower CI | Upper CI | *χ^2^* | df | p |
| --- | --- | --- | --- | --- | --- | --- | --- |
| (Intercept) | -3.27 | 0.14 | -3.54 | -3.01 |  |  | ^(1)^ |
| Condition (experimental) | 0.49 | 0.09 | 0.31 | 0.67 | 26.51 | 1 | <.001 |
| Age (18 months) | -0.06 | 0.09 | -0.24 | 0.13 | 2.13 | 1 | .145 |
| Population (UK) | -0.06 | 0.10 | -0.24 | 0.13 | 0.46 | 1 | .500 |
| Sex (male) | -0.05 | 0.09 | -0.23 | 0.13 | 0.30 | 1 | .581 |

^(1)^ H_0_ for the intercept is *intercept = 0.* The intercept represents the hypothetical value of the response assuming all predictors and random effects are zero, but the absolute baseline of the response is not of interest, therefore is not reported.

***S7b Comforting***

Results of the reduced binomial model of the effects of condition, age, population, and infant sex on infants’ probability to comfort can be found in Table S7b.

**Table S7b**

**Model Estimates with Standard Errors, Confidence Intervals, and Test Results**

| Term | Estimate | SE | Lower CI | Upper CI | *χ^2^* | df | p |
| --- | --- | --- | --- | --- | --- | --- | --- |
| (Intercept) | -4.17 | 0.61 | -6.15 | -3.24 |  |  | ^(1)^ |
| Condition (experimental) | 2.47 | 0.54 | 1.62 | 4.18 | 36.88 | 1 | <.001 |
| Age (18 months) | 0.60 | 0.37 | -0.09 | 1.44 | 2.72 | 1 | .099 |
| Population (UK) | -0.41 | 0.33 | -1.13 | 0.24 | 1.60 | 1 | .207 |
| Sex (male) | -0.21 | 0.34 | -0.97 | 0.44 | 0.39 | 1 | .535 |

^(1)^ Not indicated because of having a very limited interpretation- see Table S7a for explanation

**Age and Site Effects**

***S7c Concerned Facial Affect***

Results of the full beta model of the effects of age, population, and infant sex on the proportion visible trial time (in the experimental condition) infants spent showing concerned facial affect towards their mother can be found in Table S7c

**Table S7c**

**Model Estimates with Standard Errors, Confidence Intervals, and Test Results**

| Term | Estimate | SE | Lower CI | Upper CI | *χ^2^* | df | p |
| --- | --- | --- | --- | --- | --- | --- | --- |
| (Intercept) | -2.55 | 0.20 | -2.98 | -2.18 |  |  | ^(1)^ |
| Age (18 months) | 0.33 | 0.23 | -0.11 | 0.80 |  |  | ^(2)^ |
| Population (UK) | -0.11 | 0.22 | -0.57 | 0.29 |  |  | ^(2)^ |
| Sex (male) | -0.18 | 0.16 | -0.53 | 0.11 | 1.31 | 1 | .253 |
| Age (18 months) * population (UK) | -0.16 | 0.32 | -0.79 | 0.47 | 0.26 | 1 | .609 |

^(1)^ Not indicated because of having a very limited interpretation- see Table S7a for explanation ^(2)^ Not indicated due to involvement in interaction

***S7d Comforting***

Results of the reduced binomial model of the effects of age, population, and infant sex on infants’ probability to comfort their mother can be found in Table S7d.

**Table S7d**

**Model Estimates with Standard Errors, Confidence Intervals, and Test Results**

| Term | Estimate | SE | Lower CI | Upper CI | *χ^2^* | df | p |
| --- | --- | --- | --- | --- | --- | --- | --- |
| (Intercept) | -1.52 | 0.41 | -2.79 | -0.83 |  |  | ^(1)^ |
| Age (18 months) | 1.21 | 0.43 | 0.50 | 2.54 | 8.70 | 1 | .003 |
| Population (UK) | -0.59 | 0.41 | -1.48 | 0.19 | 2.12 | 1 | .145 |
| Sex (male) | -0.47 | 0.42 | -1.53 | 0.34 | 1.28 | 1 | .257 |

^(1)^ Not indicated because of having a very limited interpretation- see Table S7a for explanation

**Familiarity and Site Effects**

***S7e Concerned Facial Affect***

Results of the full beta model of the effects of model familiarity, population, and infant sex on the proportion of visible trial time infants spent showing concerned facial affect at 18 months) can be found in Table S7e.

**Table S7e**

**Model Estimates with Standard Errors, Confidence Intervals, and Test Results**

| Term | Estimate | SE | Lower CI | Upper CI | *χ^2^* | df | p |
| --- | --- | --- | --- | --- | --- | --- | --- |
| (Intercept) | -2.05 | 0.21 | -2.51 | -1.64 |  |  | ^(1)^ |
| Adult (mother) | 0.09 | 0.25 | -0.43 | 0.58 |  |  | ^(2)^ |
| Population (UK) | 0.08 | 0.24 | -0.39 | 0.58 |  |  | ^(2)^ |
| Sex (male) | -0.05 | 0.17 | -0.36 | 0.30 | 0.08 | 1 | .772 |
| Adult (mother) * culture (UK) | -0.33 | 0.34 | -1.03 | 0.35 | 0.93 | 1 | .335 |

^(1)^ Not indicated because of having a very limited interpretation- see Table S7a for explanation ^(2)^ Not indicated due to involvement in interaction

***S7f Comforting***

Results of the reduced binomial model of the effects of model familiarity, population, and infant sex on infants’ probability to comfort at 18 months can be found in Table S7f.

**Table S7f**

**Model Estimates with Standard Errors, Confidence Intervals, and Test Results**

| Term | Estimate | SE | Lower CI | Upper CI | *χ^2^* | df | p |
| --- | --- | --- | --- | --- | --- | --- | --- |
| (Intercept) | -1.76 | 0.47 | -14.89 | -1.05 |  |  | ^(1)^ |
| Adult (mother) | 1.50 | 0.48 | 0.69 | 9.00 | 11.44 | 1 | <.001 |
| Population (UK) | -0.55 | 0.43 | -1.66 | 0.35 | 1.63 | 1 | .202 |
| Sex (male) | -0.96 | 0.45 | -3.19 | 0.05 | 2.31 | 1 | .128 |

^(1)^ Not indicated because of having a very limited interpretation- see Table S7a for explanation

**Longitudinal Predictiveness of Early Empathy**

***S7g Does Concerned Facial Affect at 9 Months Predict Comforting at 18 Months?***

Results of the full binomial model of the effects of concerned facial affect at 9 months, population, and infant sex on infants’ probability to comfort their mother at 18 months can be found in Table S7g.

**Table S7g**

**Model Estimates with Standard Errors, Confidence Intervals, and Test Results**

| Term | Estimate | SE | Lower CI | Upper CI | *χ^2^* | df | p |
| --- | --- | --- | --- | --- | --- | --- | --- |
| (Intercept) | 0.41 | 0.49 | -0.64 | 1.52 |  |  | ^(1)^ |
| Concern (yes) | -1.76 | 5.20 | -3.84 | -0.07 |  |  | ^(2)^ |
| Population (UK) | -0.75 | 0.63 | -2.06 | 0.52 |  |  | ^(2)^ |
| Sex (male) | -1.27 | 0.69 | -2.59 | -0.10 | 4.57 | 1 | .033 |
| Concern (yes) * Population (UK) | 0.16 |  | -3.22 | 2.98 | 0.01 | 1 | .916 |

^(1)^ Not indicated because of having a very limited interpretation- see Table S7a for explanation ^(2)^ Not indicated due to involvement in interaction

**S8 Power Analysis for Significant Effects**

The table below concerns a post-hoc power analysis of all significant effects, computed using the package *simr* in R. The success column indicates the likelihood of detecting the effect of interest across 100 simulations, with 0.8 (80% of simulations) being considered the threshold for sufficient power.

**Table S8**

**Post-hoc Power Analysis of All Significant Effects**

| Research Question | Measure | Effect of interest | Success | Lower CI | Upper CI |
| --- | --- | --- | --- | --- | --- |
| Condition Effect | Concerned facial affect | Condition | 1 | 0.96 | 1 |
|  | Comforting |  | 1 | 0.96 | 1 |
|  | Hypothesis testing |  | 1 | 0.96 | 1 |
| Age and site effects | Concerned facial affect | No significant effect found | | | |
|  | Comforting | Age | 0.82 | 0.73 | 0.89 |
|  | Hypothesis testing | Age | 1 | 0.96 | 1 |
| Familiarity and site effects | Concerned facial affect | No significant effect found | | | |
|  | Comforting | Familiarity | 0.92 | 0.85 | 0.96 |
|  | Hypothesis testing | No significant effect found | | | |

**S9 Additional Bayesian Analysis**

As our sample sizes were modest and a-priori power analyses for this specific study were not appropriate (sample size constrained by overall longitudinal project), we also ran Bayesian versions of our final reduced models. Convergence in patterns of results between frequentist and Bayesian approaches can be indicative of robust findings.

**General Method**

We fitted these models using the *brms* package. Each model included four Markov chain Monte Carlo chains, with 2000 iterations per chain, of which we specified 500 iterations as warm-up to ensure sampling calibration. This resulted in 6000 posterior samples in total for each model. We used default weakly informative priors (student’s t-distribution of 3 degrees of freedom; scale parameter of 2.5), which a) allows the model to explore a broader parameter space so that no potential outcomes are excluded (necessary given the lack of previous comparable cross-cultural data) and b) improves comparability with our frequentist models. The model formulas were the same as those used in the frequentist models reported in the main text. All models had R-hat statistics close to 1 and effective samples of > 1000 and showed posterior distributions suggesting an accurate reflection of the original data. We report the 95% credible interval (CrI; the range of values an effect falls in with 95% probability) and the probability of direction (pd; the probability that the model estimate is strictly negative or positive, with pd > 97.5% probability corresponding to a frequentist p-value of 0.05, https://easystats.github.io/bayestestR/reference/p_direction.html#possible-range-of-values).

**Condition Effect**

Infants were more likely to display concerned facial affect (estimate = 0.49, SE = 0.09, 95% CrI [0.30, 0.67], pd = 1) and to comfort (estimate = 2.47, SE = 0.52, 95% CrI [1.55, 3.61], pd = 1) in the experimental than the control condition. These results are in line with those of the frequentist models.

**Table S9a**

**Model Estimates with Standard Errors, Confidence Intervals, and Test Results**

| Behavioural measure | Term | Estimate | Est. Error | 95% Credible Interval | Probability of Direction |
| --- | --- | --- | --- | --- | --- |
| Concerned facial affect | Intercept | -3.26 | 0.14 | -3.53, -3.00 | 1.00 |
|  | Condition (experimental) | 0.49 | 0.09 | 0.30, 0.67 | 1.00 |
|  | Age (18 months) | 0.14 | 0.10 | -0.05, 0.34 | 0.93 |
|  | Population (UK) | -0.06 | 0.09 | -0.24, 0.12 | 0.75 |
|  | Sex (male) | -0.05 | 0.09 | -0.24, 0.13 | 0.72 |
| Comforting | Intercept | -4.24 | 0.60 | -5.55, -3.15 | 1.00 |
|  | Condition (experimental) | 2.47 | 0.52 | 1.55, 3.61 | 1.00 |
|  | Age (18 months) | 0.61 | 0.38 | -0.11, 1.34 | 0.95 |
|  | Population (UK) | -0.42 | 0.35 | -1.11, 0.25 | 0.89 |
|  | Sex (male) | -0.21 | 0.35 | -0.90, 0.46 | 0.73 |

**Age and Site Effects**

Infants were more likely to comfort at 18 than 9 months (estimate = 0.25, SE = 0.18, 95% CrI [-0.06, 0.55], pd = 0.95). There was no difference in how likely infants from Uganda or the UK were to comfort at either age (estimate = -0.66, SE = 0.45, 95% CrI [-1.59, 0.22], pd = 0.93), and neither age (estimate = 0.25, SE = 0.16, 95% CrI [-0.06, 0.55], pd = 0.94) nor population (estimate = -0.19, SE = 0.16, 95% CrI [-0.49, 0.12], pd = 0.88) affected the relative amount of displayed concerned facial affect. These results are in line with those of the frequentist models.

**Table S9b**

**Model Estimates with Standard Errors, Confidence Intervals, and Test Results**

| Behavioural measure | Term | Estimate | Est. Error | 95% Credible Interval | Probability of Direction |
| --- | --- | --- | --- | --- | --- |
| Concerned facial affect | Intercept | -2.50 | 0.18 | -2.86, -2.15 | 1.00 |
|  | Age (18 months) | 0.25 | 0.16 | -0.06, 0.55 | 0.94 |
|  | Population (UK) | -0.19 | 0.16 | -0.49, 0.12 | 0.88 |
|  | Sex (male) | -0.18 | 0.16 | -0.50, 0.13 | 0.87 |
| Comforting | Intercept | -1.68 | 0.47 | -2.65, -0.83 | 1.00 |
|  | Age (18 months) | 1.28 | 0.45 | 0.44, 2.18 | 1.00 |
|  | Population (UK) | -0.66 | 0.45 | -1.59, 0.22 | 0.93 |
|  | Sex (male) | -0.49 | 0.46 | -1.43, 0.38 | 0.86 |

**Familiarity and Site Effects**

Infants were more likely to comfort their mother than an experimenter at 18 months (estimate = 1.61, SE = 0.51, 95% CrI [0.66, 2.65], pd = 1). There was no difference in how likely infants from Uganda or the UK were to comfort either adult (estimate = -0.59, SE = 0.49, 95% CrI [-1.59, 0.33], pd = 0.89), and neither adult familiarity (estimate = -0.09, SE = 0.17, 95% CrI [-0.43, 0.25], pd = 0.70) nor population (estimate = -0.08, SE = 0.17, 95% CrI [-0.43, 0.26], pd = 0.68) affected the relative amount of displayed concerned facial affect. These results are in line with those of the frequentist models.

**Table S9c**

**Model Estimates with Standard Errors, Confidence Intervals, and Test Results**

| Behavioural measure | Term | Estimate | Est. Error | 95% Credible Interval | Probability of direction |
| --- | --- | --- | --- | --- | --- |
| Concerned facial affect | Intercept | -1.95 | 0.18 | -1.60, 1.00 | 100.00% |
|  | Adult (mother) | -0.09 | 0.17 | -0.43, 0.25 | 69.93% |
|  | Population (UK) | -0.08 | 0.17 | -0.43, 0.26 | 68.37% |
|  | Sex (male) | -0.04 | 0.17 | -0.39, 0.29 | 59.85% |
| Comforting | Intercept | -1.94 | 0.54 | -3.13, -0.98 | 100.00% |
|  | Adult (mother) | 1.61 | 0.51 | 0.66, 2.65 | 99.97% |
|  | Population (UK) | -0.59 | 0.49 | -1.59, 0.33 | 89.42% |
|  | Sex (male) | -0.73 | 0.50 | -1.72, 0.20 | 93.58% |
